# PROSPECTIVE EXPLORATION OF COMBINED INTERNALIZING SYMPTOMS ON SELF-REPORTED MEMORY DURING COVID-19

**DOI:** 10.1093/geroni/igad104.2626

**Published:** 2023-12-21

**Authors:** Dawn Carr, Natalie Sachs-Ericsson, Brad Schmidt, Frederick Schubert

**Affiliations:** Florida State University, Tallahassee, Florida, United States; Florida State University, Tallahassee, Florida, United States; Florida State University, Tallahassee, Florida, United States; Florida State University, Tallahassee, Florida, United States

## Abstract

A growing literature suggests depression and anxiety increase risk of cognitive decline. However, few studies have examined their combined effects on cognition, among older adults, especially during periods of high stress. Based on a sample of community dwelling older adults (N=576), we evaluated the effects of pre-pandemic anxiety and depressive symptoms, obtained in September 2018, to changes in self-reported memory (SRM) assessed 3 months into the COVID-19 pandemic. In separate models we found participants with depression scores at least 1-SD above the mean and participants with anxiety scores at least 2-SD above the mean to report a significant decline in SRM. Moderation analyses revealed those with high depressive symptoms (at or above the mean) showed a decrease in SRM regardless of anxiety. The extent to which high pre-pandemic anxiety symptoms influenced SRM is dependent on whether pre-pandemic depression was at or above the mean. Pre-pandemic depression predicted a decline in SRM regardless of anxiety. Moderation analyses revealed that the extent to which anxiety symptoms influenced SRM was dependent on depression being at or above the mean. Those with high anxiety and depression are at highest risk of experiencing cognitive consequences related to stressful exposures like COVID-19.

